# Broadband Access and Ophthalmologist Density in the United States: Cross-Sectional Questionnaire Study

**DOI:** 10.2196/88473

**Published:** 2026-05-19

**Authors:** Patrick Akarapimand, John C Lin, Ingrid U Scott, Sally L Baxter

**Affiliations:** 1 Department of Ophthalmology, Scheie Eye Institute, Perelman School of Medicine, University of Pennsylvania, Philadelphia, PA, United States; 2 Department of Ophthalmology, Pennsylvania State College of Medicine, Pennsylvania State University, Hershey, PA, United States; 3 Department of Public Health Sciences, Pennsylvania State College of Medicine, Pennsylvania State University, Hershey, PA, United States; 4 Hamilton Glaucoma Center and Division of Ophthalmology Informatics and Data Science, Viterbi Family Department of Ophthalmology and Shiley Eye Institute, University of California San Diego, 9415 Campus Point Drive, MC 0946, La Jolla, CA, 92093, United States, 1 858-534-6290; 5 Division of Biomedical Informatics, Department of Medicine, University of California San Diego

**Keywords:** broadband, teleophthalmology, ophthalmologist density, rural urban divide, public health

## Abstract

**Background:**

Rural US communities experience disproportionately high rates of visual disability yet have limited access to ophthalmologists. Teleophthalmology may help address these gaps, but its effectiveness depends on broadband connectivity. The relationship between broadband access and ophthalmologist density has not been well characterized.

**Objective:**

The aim of this study is to quantify the association between household broadband access—defined as subscription rates or connection prevalence—and county-level ophthalmologist density and to identify sociodemographic predictors of access.

**Methods:**

We conducted an ecological study of all 3141 US counties using 2019 data from the American Community Survey, Area Health Resources File, and National Center for Health Statistics (NCHS). Broadband access was the primary exposure; ophthalmologist count with county population as an offset was the outcome. The primary analysis used negative binomial regression, adjusting for urbanicity, income, education, age, sex, race/ethnicity, unemployment, and insurance status. Sensitivity analyses included population-weighted linear regression and state fixed effects models. County-level heatmaps illustrated geographic patterns.

**Results:**

Median household broadband access was 56.6%, ranging from 72.2% in the most urban counties (NCHS category 1) to 49.1% in the most rural (NCHS category 6). In unadjusted negative binomial regression, each 10-percentage-point increase in broadband access was associated with a 68% higher ophthalmologist rate (incidence rate ratio=1.68, 95% CI 1.61‐1.76; *P*<.001). After adjustment, each 10-percentage-point increase was associated with a 46% higher rate (incidence rate ratio=1.46, 95% CI 1.37‐1.56; *P*<.001). Sensitivity analyses were consistent with primary analysis. Regions with both low broadband access and zero ophthalmologist density were concentrated in the South, Mountain West region, and Alaska.

**Conclusions:**

Broadband access is strongly associated with ophthalmologist availability across US counties, independent of sociodemographic factors. Areas lacking ophthalmologists also tend to lack broadband adoption, creating compounded barriers to both in-person and teleophthalmic care. Efforts to expand broadband may support more equitable access to vision services in underserved regions.

## Introduction

Roughly 20% of the US population lives in a rural setting; those living in rural settings are more likely than those living in urban setting to experience disability [1]. Of those experiencing disability, visual disability in particular is highly prevalent among adults aged 18 years or older, with recent estimates approximating that 7 million people in the United States live with visual impairment as defined by visual acuity loss [2,3]. Although 90% of people with visual impairment have preventable or treatable conditions [4], areas with limited or no access to local eye care providers pose a significant concern in the United States [5,6]. This disparity is particularly concentrated in rural areas, where there are lower densities of ophthalmologists and patients are unable to travel the distance required to attend appointments [5,6].

Improving access to eye care in rural areas remains a key health policy; however, many obstacles exist for increasing provider density, access, and funding in these areas. Currently, only 11% of physicians practice in rural areas [7]. This lack of physicians is exacerbated by estimates that predict a nearly 25% reduction in the rural physician workforce as many rural physicians near retirement [8]. The effort to draw younger physicians to rural settings is complicated by fewer opportunities for spousal employment, more limited school resources for their children, and the financial disincentive of a lower income in the face of mounting student debt [7,9,10]. The idea of specialization in ophthalmology is also in particular discordance with the notion that rural physicians must practice more broadly due to fewer health care resources [11,12].

During the height of the COVID-19 pandemic, ambulatory outpatient visits declined by 79% [13], with studies showing lasting reductions in ophthalmic care utilization [14]. As an answer to the physical limitations of pandemic lockdown orders and national policies, teleophthalmology gained unprecedented attention and adoption on a large scale [15]. This growth of interest in teleophthalmology as a potential solution for otherwise geographically limited vision services also highlights the importance of broadband internet access, which is crucial for many components of teleophthalmology visits such as synchronous video with audio and asynchronous messaging and sharing of vital health information between patient and provider [16]. Beyond the primary connection that broadband access enables, it also connects patients to online resources containing vision health information [17].

Given the linkage of broadband access and teleophthalmology services, broadband access may be a useful target to improve access to vision care, particularly for rural populations who otherwise lack access to providers. However, to our knowledge, broadband access has not been studied in association with access to physical ophthalmic facilities, personnel, and services. Additionally, the intersection of broadband access across geographical distributions and other social determinants of health is poorly understood. Exploring this association may help inform public interventions to increase equity in broadband access and access to ophthalmic services. Therefore, we utilized data from the Area Health Resource Files, National Center for Health Statistics (NCHS), and American Community Survey (ACS) to assess for associations between disparities in broadband internet access and access to ophthalmology specialists.

## Methods

### Ethical Considerations

This study was considered exempt from full review per the University of Pennsylvania Institutional Review Board guidelines [18], as it used only deidentified, publicly available, aggregate county-level data and did not involve human subjects research requiring informed consent. The study adhered to the principles of the Declaration of Helsinki. No individual-level patient data were accessed or collected. All data were obtained from 2019 versions of each dataset.

### Study Design

The number of practicing ophthalmologists per county and unemployment rates were obtained from the Area Health Resource Files. Urbanicity was categorized according to the NCHS 6-level urban–rural classification scheme (1=large central metro, 2=large fringe metro, 3=medium metro, 4=small metro, 5=micropolitan, and 6=noncore). This approach avoids the assumption of a linear step relationship between categories that would be imposed by treating the codes as continuous. All other demographic and socioeconomic variables were obtained from ACS, including median household income, percentage of the population who were female, Black, Hispanic, aged ≥65 years, with less than a high school education, and uninsured. Case complete analysis was performed with 91 counties omitted for missing data.

The primary exposure was broadband access, defined as the percentage of households in a county with broadband internet access such as cable, fiber optic, or DSL. This measure reflects the proportion of households with functional internet connectivity, rather than direct availability of broadband infrastructure, speed, or affordability. Broadband access was treated as a continuous variable and adjusted per 10% for interpretability. The primary outcome was the number of ophthalmologists per county. County population size was incorporated as an offset term to model ophthalmologist availability relative to county population.

Descriptive statistics were used to summarize broadband access across all counties. Bivariate associations between broadband access and ophthalmologist count were initially assessed using simple linear regression. Because the outcome variable represented a count of ophthalmologists and demonstrated overdispersion, the primary analysis used negative binomial regression to estimate the association between broadband access and the number of ophthalmologists per county. County population size was included as an offset term to model ophthalmologist supply relative to population. The multivariable model adjusted for potential county-level confounders including urbanicity, median household income, and percentage of the population who were female, Black, Hispanic, aged ≥65 years, with less than a high school education, unemployed, and uninsured. Incidence rate ratios (IRR) were then calculated. To assess potential multicollinearity among predictors, variance inflation factors were calculated for all independent variables.

Sensitivity analyses were conducted to evaluate the robustness of the findings. First, a population-weighted multiple linear regression model was fitted with ophthalmologist density (ophthalmologists per 100,000 population) as the outcome, weighting counties by population size to reduce the influence of sparsely populated counties. Second, models incorporating state fixed effects were estimated to control for unobserved state-level factors, such as regulatory environments, telehealth policy, and workforce training infrastructure.

County-level geographic boundaries were obtained from the US Census Bureau’s 2019 TIGER/Line Shapefiles [19]. Broadband access and ophthalmologist density, adjusted into unique deciles for interpretability, were then mapped to US counties. Additional heatmaps identified counties with low broadband access (defined as <1st quartile) and low ophthalmologist density (defined as 0 ophthalmologists per 100,000 people). We also conducted further exploratory analysis to investigate whether household broadband access varies systematically across county socioeconomic characteristics. All analyses and visualizations were conducted using RStudio (version 4.4.0; Posit).

## Results

Data from 3141 US counties were included. Median broadband access across all counties was 56.6%. In the most urban counties (NCHS category 1), 72.2% of households had broadband, and there were 9.1 ophthalmologists per 100,000 people, whereas the most rural counties (NCHS category 6) had 49.1% of households with broadband and 0.43 ophthalmologists per 100,000.

Bivariate analysis showed a significant association between broadband access and ophthalmologist counts, where each 10-percentage-point increase in household broadband access was associated with a 68% higher rate of ophthalmologists per county (IRR=1.68, 95% CI 1.61‐1.76; *P*<.001). After adjusting for urbanicity, income, education, age, sex, race/ethnicity, unemployment, and insurance status, each 10-percentage-point increase in broadband access was associated with a 46% higher ophthalmologist supply per capita (IRR 1.46, 95% CI 1.37‐1.56; *P*<.001, Table 1).

**Table 1.** Negative binomial regression of ophthalmologist counts on broadband access and covariates.

Variable	Incidence rate ratio	95% CI	*P* value
Household broadband access (per 10%)	1.46	1.37‐1.56	<.001
NCHS category 2 (large fringe metro)	0.47	0.39‐0.55	<.001
NCHS category 3 (medium metro)	0.82	0.69‐0.98	.03
NCHS category 4 (small metro)	0.99	0.82‐1.19	.93
NCHS category 5 (micropolitan)	0.85	0.7‐1.04	.12
NCHS category 6 (noncore)	0.32	0.25‐0.42	<.001
Median household income (per $10,000)	0.99	0.95‐1.03	.54
Less than high school education (%)	0.96	0.94‐0.97	<.001
Age ≥65 (%)	1.01	0.99‐1.02	.35
Female (%)	1.21	1.16‐1.26	<.001
Black (%)	1.02	1.01‐1.02	<.001
Hispanic (%)	1.01	1.01‐1.02	<.001
Unemployment rate (%)	0.94	0.90‐0.98	.005
Uninsured (%)	0.99	0.97‐1.00	.08

In multiple linear regression, we assessed ophthalmologist density and broadband access after adjusting for urbanicity (NCHS Urban-Rural Classification), median household income (per $10,000), and percentage of the population who were female, Black, Hispanic, aged 65 years and older, with less than a high school education, unemployed, and uninsured.

Compared with large central metropolitan counties, noncore counties had a 68% lower ophthalmologist availability (IRR 0.32, 95% CI 0.25‐0.42; *P*<.001). Variance inflation factors for all predictors were below 2, indicating minimal multicollinearity.

Population-weighted linear regression produced consistent findings. Each 10-percentage-point increase in broadband access was associated with 0.43 additional ophthalmologists per 100,000 population ((*β*=.43, 95% CI 0.18‐0.68; *P*<.001). Furthermore, models including state fixed effects demonstrated a persistent association (IRR 1.63, 95% CI 1.51‐1.76; *P*<.001), suggesting that the relationship was present even accounting for unobserved state-level factors.

**Figure 1.**
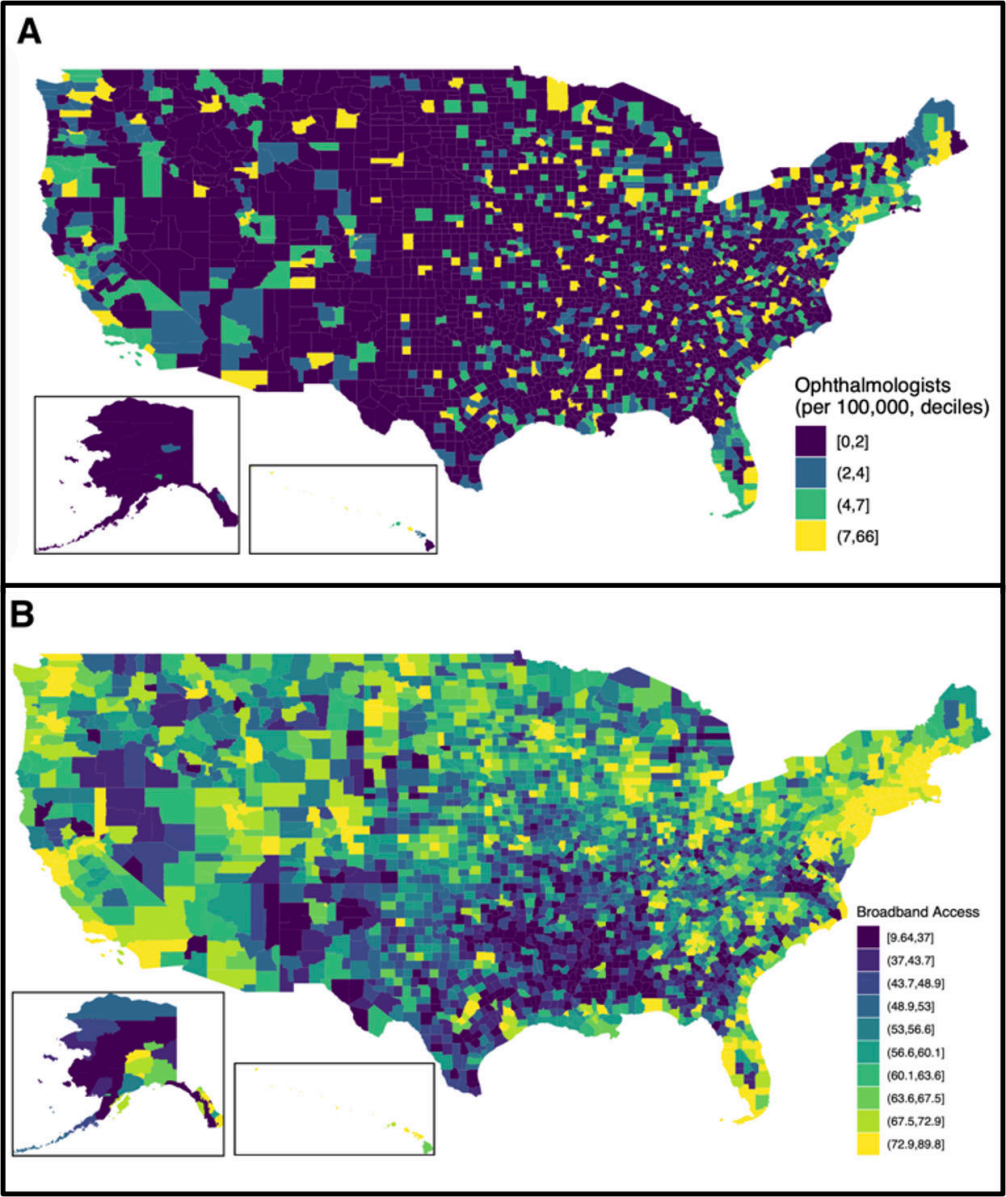
Heatmaps of ophthalmologist density (Panel A) and broadband access (Panel B) by US county, 2019.

**Figure 2.**
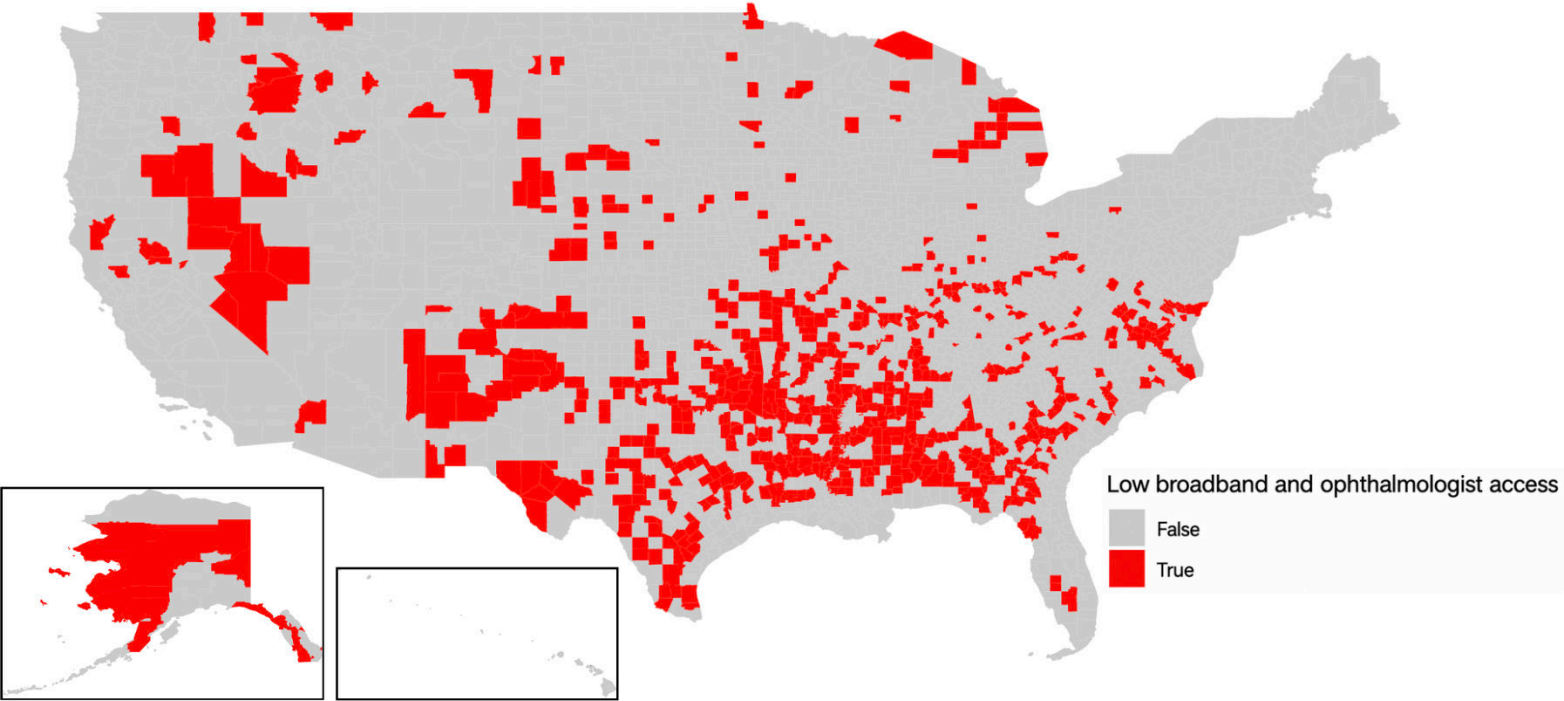
Heatmap of US counties with both low broadband access and low ophthalmologist access, 2019. Defined by broadband access <1st quartile (46.27%) and ophthalmologist density of 0 per 100,000 people. In total, 700 counties met these criteria, encompassing approximately 11.4 million residents. The heatmap demonstrates concentration in the South, Mountain West, and Alaska regions.

Figure 1 illustrates geographic variation in ophthalmologist density and broadband access by US counties. Figure 2 illustrates that counties with both low broadband access and ophthalmologist density were concentrated in the South, Mountain West, and Alaska regions.

**Figure 3.**
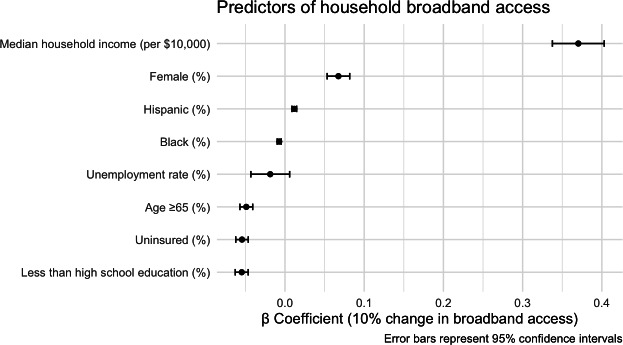
Forest plot showing *β* coefficients and 95% CIs from multiple linear regression of household broadband access among socioeconomic covariates. Results show that higher proportions of females and Hispanic people, as well as higher median household income, are positively associated with broadband access, whereas high proportions of Black people, adults aged 65 or older, uninsured people, and those with no high school education were negatively associated with broadband access.

In multivariable linear regression, several sociodemographic characteristics were associated with household broadband access across the United States. Lower educational attainment (*β*=−0.05, 95% CI −0.06 to −0.05), higher proportions of adults ≥65 years (*β*=−0.05, 95% CI −0.06 to −0.05), higher percentages of Black residents (*β*=−0.007, 95% CI −0.010 to −0.005), and higher percentages of uninsured residents (*β*=−0.05, 95% CI −0.06 to −0.05) were associated with lower broadband access. Conversely, higher median household income (*β*=0.37, 95% CI 0.34 to 0.40), greater proportions of female residents (*β*=0.07, 95% CI 0.05 to 0.08), and higher percentages of Hispanic residents (*β*=0.01, 95% CI 0.009 to 0.015) were positively associated with access. Unemployment showed a marginal negative association that did not reach significance (*β*=−0.02, 95% CI −0.04 to 0.006). Although several predictors reached statistical significance, the effect sizes were modest. Results are visualized in Figure 3.

## Discussion

Teleophthalmology has been suggested as an innovative solution to improve access to ophthalmic services in communities with fewer vision care resources. However, broadband access and adoption to enable the full functionalities of teleophthalmology in these areas has not been well studied. In this study assessing broadband access with ophthalmologist density in 3141 US counties, we found that US counties with reduced broadband access also have lower average counts and densities of ophthalmologists. Importantly, the association between broadband access and ophthalmologist supply remained robust across multiple sensitivity analyses designed to address potential methodological concerns. Results were consistent after population-weighted modeling and incorporating state fixed effects, suggesting that the observed relationship is not driven by small population counties or unmeasured state-level factors, strengthening the inference that broadband access and ophthalmologist distribution are closely linked structural features of health care access. This finding underscores that while teleophthalmology may mitigate key barriers to in-person vision care, lack of broadband access may present an additional, underexplored barrier to care for individuals across the spectrum of urbanicity. These findings may be particularly concentrated among rural regions in the South, Mountain West region, and Alaska. Furthermore, in secondary analysis, we found that broadband access tends to be independently lower in communities that are more rural, have lower levels of educational attainment, and higher proportions of older adults over 65, Black residents, and uninsured individuals, although with modest effect sizes.

An important interpretive consideration is that the ACS broadband measure used in this study captures household subscription rates—reflecting whether households have an active broadband connection—rather than physical infrastructure availability, service speeds, or affordability. A county may have fiber infrastructure yet show low subscription rates due to cost barriers, limited digital literacy, or a lack of devices. Conversely, a county may have high adoption rates despite suboptimal infrastructure due to demographic and socioeconomic characteristics. Therefore, our findings should be interpreted as demonstrating an association between low broadband adoption and low ophthalmologist density, rather than between infrastructure deficits and provider scarcity per se. Efforts to improve broadband equity must therefore address both availability (deployment of physical infrastructure) and adoption (affordability subsidies, device access programs, and digital literacy training), as low adoption can persist even after infrastructure gaps are closed.

Our findings align with current literature demonstrating sociodemographic disparities in broadband access. For instance, several studies suggest that areas with larger proportions of Black and American Indian/Alaska Native populations, lower economic status, and lower educational attainment tend to have less broadband access [20,21]. Our results also further support several studies demonstrating disproportionately limited access to ophthalmic care in rural areas and communities with lower socioeconomic status [22-24]. Our study adds to the current literature that the association between household broadband access and ophthalmologist density persists across the urban/rural divide. This means that even in metropolitan counties where in-person care is relatively accessible, low broadband subscription may independently limit the reach of supplemental teleophthalmology services. This is exacerbated in nonmetropolitan areas, where even fewer ophthalmologists are available and there is poorer broadband access. This study thus expands on the current literature by directly contextualizing broadband access within the broader scope of ophthalmology access, showing that areas void of physical providers also lack the connectivity necessary for remote solutions, creating compounded access barriers.

The clear association between broadband access and socioeconomic factors raises the concern that broadband expansion alone may not lead to meaningful increases in health care utilization. Digital literacy gaps, especially among older adults, disabled people, or low-income populations, may persist independent of connectivity. Indeed, studies have found that older adults and individuals with lower education levels may be less likely to engage with online health tools or complete telehealth visits due to unfamiliarity with technology or distrust in virtual care models [25-29]. Others have argued that teleophthalmology is limited in clinical scope for procedures requiring in-person physical exams, diagnostic imaging, or surgical interventions [30-33]. Another critique is based on cost-effectiveness: broadband infrastructure costs are particularly high in geographically isolated regions, where population density may be too low to justify investment on health care grounds alone [34-36].

However, these concerns, while valid, do not negate the importance of broadband as a foundational infrastructure for equitable health care delivery. First, digital literacy gaps are surmountable with targeted interventions. Programs that pair broadband rollouts with community-based digital navigator training have successfully improved technology adoption in older adults, racial/ethnic minorities, and low-income groups [37-40]. Second, teleophthalmology has evolved beyond synchronous video visits to include asynchronous image-based screening, such as store-and-forward models for diabetic retinopathy and glaucoma, which require minimal digital interaction by the patient and are well-suited for underserved communities [30,41-45]. For instance, Google/Verily’s ARDA partnership with Aravind Eye Care in India demonstrates that clinic-based broadband can mitigate household limitations [46]. Nevertheless, widespread household connectivity may enhance patient participation in remote screening and education.

Federal efforts such as the Broadband Equity, Access, and Deployment program, part of the 2021 Infrastructure Investment and Jobs Act, already allocate billions of dollars toward rural broadband with explicit equity goals, aligning policy momentum with the needs demonstrated in this study [47]. Further interventions include the expansion of federal programs such as the US Department of Agriculture Rural eConnectivity Pilot Program [48], including the removal of eligibility restrictions to allow all rural areas with inadequate broadband access to apply for funding. Furthermore, telehealth policy reform, such as reimbursement parity and interstate licensure compacts, will further support teleophthalmology and eye care access in underserved regions [34,49]. Our results provide empirical evidence that leveraging policy funding to support prioritizing broadband investments, particularly in the rural South, Mountain West region, and Alaska and in regions with lower educational attainment and higher proportions of older adults, Black people, and uninsured people, may be particularly high yield for both technological and health system gains.

This study contains a few key limitations. First, the ACS broadband measure captures household subscription rates, not infrastructure availability, speed, latency, or affordability, and therefore cannot distinguish between cost barriers, digital literacy gaps, device limitations, and physical infrastructure deficits as drivers of low broadband access. Furthermore, our data only consider the percentage of households that lack access to broadband internet. As a result, access to broadband outside the household in facilities such as workplaces, schools, or public facilities remains unclear. However, transportation, lack of convenience, and privacy concerns arising from utilizing broadband access outside of one’s home may present as barriers; therefore, accessibility to household broadband internet is still relevant. In addition, our data were obtained from 2019, which was before the massive expansion in broadband internet access in response to the COVID-19 pandemic. Lastly, this ecological, cross-sectional study cannot establish causation or temporal sequence, and unmeasured county-level variables may confound associations, as is the case with all cross-sectional studies.

To our knowledge, this study represents the first national analysis examining the relationship between broadband access and ophthalmologist workforce distribution across all US counties. These two factors appear to be intertwined and may reinforce each other in determining rural and underserved communities’ connectivity to vision care. As teleophthalmology becomes integral to future eye care delivery, advancing broadband equity should be positioned as a public health strategy that simultaneously bolsters virtual and in-person specialist access.

## Supplementary material

10.2196/88473 Multimedia Appendix 1 Dataset.

## Abbreviations

ACS: American Community Survey
IRR: incidence rate ratio
NCHS: National Center for Health Statistics
